# Precision of Fit of Titanium and Cast Implant Frameworks Using a New Matching Formula

**DOI:** 10.1155/2012/374315

**Published:** 2012-04-08

**Authors:** Marianella Sierraalta, Jose L. Vivas, Michael E. Razzoog, Rui-Feng Wang

**Affiliations:** ^1^Division of Prosthodontics, Department of Biologic and Material Science, The University of Michigan, School of Dentistry, 1011 North University Avenue, Ann Arbor, MI 48109-1078, USA; ^2^Department of Cariology, Restorative Sciences, and Endodontics, The University of Michigan, School of Dentistry, Ann Arbor, MI 48109-1078, USA

## Abstract

*Statement of the Problem*. Fit of prosthodontic frameworks is linked to the lifetime survival of dental implants and maintenance of surrounding bone. *Purpose*. The purpose of this study was to evaluate and compare the precision of fit of milled one-piece Titanium fixed complete denture frameworks to that of conventional cast frameworks. *Material and Methods*. Fifteen casts fabricated from a single edentulous CAD/CAM surgical guide were separated in two groups and resin patterns simulating the framework for a fixed complete denture developed. Five casts were sent to dental laboratories to invest, cast in a Palladium-Gold alloy and fit the framework. Ten casts had the resin pattern scanned for fabrication of milled bars in Titanium. Using measuring software, positions of implant replicas in the definitive model were recorded. The three dimensional spatial orientation of each implant replica was matched to the implant replica. *Results*. Results demonstrated the mean vertical gap of the Cast framework was 0.021 (+0.004) mm and 0.012 (0.002) mm determined by fixed and unfixed best-fit matching coordinate system. For Titanium frameworks they were 0.0037 (+0.0028) mm and 0.0024 (+0.0005) mm, respectively. *Conclusions*. Milled one-piece Titanium fixed complete denture frameworks provided a more accurate precision of fit then traditional cast frameworks.

## 1. Introduction

Osseointegrated dental implants have been proven successful in the treatment of edentulism [1]. Several techniques have been described for the successful restoration of the edentulous ridges, one being the fixed complete dentures [2]. Among the procedures used in the fabrication of those prostheses is the milled bar [3]. 

Meanwhile numerous articles emphasize the importance of passivity of implant-prosthetic component interfaces [4–6]. A nonpassive interface between the mating surface of the framework to its intended interface position to the implants or abutments has been implicated as a causative factor associated with implant/bone surface contact, implant screw loosening/fracture, abutment screw loosening/facture, and/or prosthetic screw loosening/fracture for abutment-based framework designs [7–9]. The conventional laboratory procedures for framework fabrication with the lost wax-casting technique are most commonly accomplished in either one piece or in segmental castings that are subsequently indexed and soldered. Discrepancies in the passive fit to its supporting abutments are occasionally encountered during the clinical try-in and evaluation appointment [10]. Frequently, the framework must then be sectioned, related in the mouth, and meticulously soldered to achieve a more accurate seating of the prosthesis to the implants. Clinically the final implant frameworks usually provide a less than absolute passive fit [11]. Nevertheless, the clinical results of applications of advanced laboratory technology to improve framework fit seem promising [12]. One of the most recent approaches to the problem of misfit is the introduction of the computer-aided design/computer-aided machined (CAD/CAM) milled one-piece titanium framework. This technique utilizes the biocompatible and relatively low-cost titanium metal and the potential for a lower risk of oral corrosion than other alloys used for implant frameworks. Further, the CAD/CAM fabrication process is less dependent on manual laboratory procedures compared to conventional casting protocols. By using an industrial manufacturing protocol for the frameworks, many factors related to manual handling of the conventional cast frameworks are controlled and avoided [13, 14].

The present investigation evaluated and compared the precision of fit of CAD/CAM one-piece titanium-fixed complete denture frameworks to that of conventional cast frameworks. Using a measurement system reported first by Jemt et al. the center point is projected in a position that is perpendicular to the component plane as the centroid point of the abutment replica [15]. This method was repeated in 2 other studies which compared the precision of fit of several types of milled fixed partial denture frameworks [16, 17]. During the present study the center point was used to determine the angular gap at the implant bar interface. The null hypothesis was that there is no differenced in the precision of interface fit between the one-piece titanium-fixed complete denture frameworks and the conventional cast frameworks.

## 2. Methods and Materials

Fifteen gypsum (Jade Stone, Whip Mix, Louisville, KY, USA) definitive casts were fabricated from a single completely edentulous surgical guide that had been designed for guided placement of six implants. While there are several different methods used to fabricate surgical guides for implant placement this study used a surgical guide fabricated using the NobelGuide (Nobel Biocare, Gothenburg, Sweden) technology. Utilizing a computer tomography (CT) of a given patient; the data obtained was then converted with the help of the software. A three-dimensional image of the bone was acquired and with this information the implants were virtually placed and the resulting surgical guide was sent for fabrication using stereolithography [18]. Using a single surgical guide, the definitive casts were fabricated.

New implant replicas as provided from the manufacturer (Nobel Replace Regular Platform, Nobel Biocare) were screwed into the guided cylinders and pins (Nobel Biocare) to ensure the geometrical relation between the guided sleeves (Nobel Biocare) and the replicas. Soft-tissue replica silicone material (Gingifast Rigid, Zhermack Spa, Badia Polesine, Italy) was added around the implant replicas and the guided sleeves. Boxing wax (Kerr Dental Laboratory Products, Orange, CA, USA) was adapted around the periphery of the surgical guide and die stone (Jade Stone, Whip Mix, Louisville, KY, USA) was mixed, following the manufacturer's guidelines in a vacuum mixer (Whip Mix, Louisville, KY, USA) and vibrated into the guide. After the stone was set, the guided cylinders with pins were removed using a Unigrip (Nobel Biocare) screwdriver, the surgical guide was separated from the definitive cast and the soft-tissue replica removed from the cast. The casts were then separated into two groups, and identical patterns to simulate the framework of a fixed complete denture (to implant level) were developed. While the measurement of the titanium frameworks was the central focus of this study, it was felt that a comparison to cast frameworks would be of some interest. The difficultly in directly comparing the cast with milled framework is that there can never be reliable controls for the fabrication of the cast framework. Thus, the intent was to obtain what a laboratory considered to be an accurate cast framework. Group A consisted of five casts, which were each sent to dental laboratories. The dental laboratories were instructed to take the patterns, invest, and then cast them in a Palladium 12%-Gold 75.1% alloy (Argedent 75, Argen Corp., San Diego, CA, USA). The laboratories were asked to use their preferred technique to obtain the optimum fit of the casting to the provided model (Test1: Cast). Group B consisted of ten casts where a resin pattern (GC Pattern Resin, GC America, Alsip, IL, USA) had been fabricated to simulate the framework of a fixed complete denture to the implant level. The single pattern was scanned for the milled framework using the NobelProcera Forte (Nobel Biocare), and the data sent to the production facility (Nobel Biocare, Mahwah, NJ, USA) for fabrication of the milled bar in titanium. Using the scanned data, a solid block of titanium was milled to produce a copy of the pattern (Test2: titanium).

Using a Zeiss Coordinate Measuring Machine and CALYPSO measuring software, positions of 6 implant replicas in the gypsum definitive model were measured and calculated as to their three dimensional spatial orientation. Positions were then matched to the measured positions of 6 framework cylinders of the corresponding cast and titanium bars (Test).

Each sample framework was measured three times. From the 450 data points of each abutment-bearing surface and mating component the centroid was computed as the 3D center. The mean of these readings was used for the statistical analysis. The centroid method achieved the best fit between the definitive cast and the test framework by achieving the total minimum vertical distance of 6 abutment/component centroids.

The centroids of one of the 6 components of a paired definitive cast/framework tested were chosen to be the matching coordinate system origin (0,0, 0) for best-fit matching. The centroid vertical gap angle between the abutment of the definitive cast and the corresponding surface of the test framework was determined for each component. The 2nd component demonstrating the minimum vertical centroid gap angle among the rest of the implant replicas was then specified. The definitive casts and test frameworks were individually rotated both horizontally and vertically, and the centroid horizontal/vertical gap angles of the 2nd component were adjusted to zero (*x*, 0,0), thus specifying the *X*-axis of the matching coordinate system. Accordingly the component demonstrating the 3rd minimum vertical centroid gap angle was found and vertically rotated as zero (*x*, *y*, 0), thus specifying the *XY*-plane of the matching coordinate system. After these calculations the centroid vertical distances of 3 out of 6 paired components become zero (0), thus the total vertical distance of the rest of 3 paired components represents the best-fit matching result between the definitive cast and the framework with the selected component as the matching coordinate system origin. Using the same procedure, but systematically assigning another centroid as the matching coordinate system origin (0,0, 0) a total of 6 matching cases were generated from each paired definitive cast/framework. The most precise best-fit case is selected from these 6 matching cases of each paired data generated by total of 120 possible matching coordinate systems (6 origins × 5 *X*-axes × 4 *XY*-planes). For statistical analysis all master data selected with such unfixed best-fit matching coordinate system method were then converted into a one fixed coordinate system numbers 1 (0,0, 0), 6 (*x*, 0,0), and 3 (*x*, *y*, 0). The test data was also transformed according to the paired master data.

The best-fit results generated by the unfixed best-fit matching coordinate system method was compared with the best-fit result generated by the fixed best-fit matching coordinate system method, where the number 1 component of each paired definitive cast/framework is the matching coordinate origin (0,0, 0), the *X*-axis of the coordinate system is always through number 6 components (*x*, 0,0), and the *XY*-plane lies on number 3 components (*x*, *y*, 0)

## 3. Results

Table 1 presents the best-fit matching results of 5 casted/definitive framework pairs generated by the unfixed coordinate system method. As explained in the methods section the unfixed coordinate system method places the frameworks onto the master cast at the point where the one set of centroids merge. The number in the first column in Table 1 indicates the specimen pair numbers 1 through 5. The matching coordinate system origin (0,0, 0) of each specimen pair was systematically designated at the centroid of component numbers 1 through 6 as demonstrated in column 2 of Table 1 so that there are total of 6 matching results generated from each measuring data of a paired framework. For instance when number 1 component centroid of the number 1 specimen pair was designated as the origin of the best-fit matching coordinate system, the centroid vertical distance between definitive and test framework was found to be minimum at number 2 component among numbers 2, 3, 4, 5, and 6 component as demonstrated in column 3 of Table 1. Then number 6 component centroid demonstrated the minimum distance between definitive and test framework among numbers 3, 4, 5, and 6 component as demonstrated in column 4 of Table 1. In this manner the *XY*-plane of the best-fit matching coordinate system were at numbers 1, 2 and 6 component centroids. The matching result in Table 1 represents the sum of the centroid vertical distance at numbers 3, 4, and 5 components (0.0126 mm for the number 1 specimen pair with number 1 component centroid as its best-fit matching coordinate system origin). Accordingly as demonstrated in the 2nd through the 5th rows when numbers 2, 3, 4, 5, and 6 component centroid was selected as the best-fit matching coordinate system origin, the matching result was 0.0058, 0.0444, 0.0527, 0.0076, and 0.0058 mm, respectively. The best-fit matching of number 1 specimen pair of definitive/cast framework was achieved when numbers 2 or 6 component centroids were designated as the best-fit matching coordinate origin, and the total centroid vertical distance of 6 components for both cases was 0.0058 mm. The same procedure was applied for each pair of definitive/cast framework as demonstrated in Table 1.  Table 2 presents the best-fit matching coordinate systems of numbers 2 through 5 definitive/casted framework pairs and their best-fit results.  Table 2 demonstrates the best-fit coordinate origin of numbers 1 through 5 paired specimen was the centroid located at the numbers 2, 2, 6, 4, and 2 components, and their best-fit results were 0.058, 0.0193, 0.0128, 0.0172 and 0.0121 mm, respectively.  Table 3 shows the best-fit matching with the unfixed coordinate system method for each pair of 10 titanium frameworks/casts.

 Tables 4 and 5 present the Test (cast or titanium) centroid best-fit locations of each component in *X*, *Y*, and *Z* axes from the corresponding master component centroid generated with the unfixed best-fit matching coordinate system.

Table 6 presents the mean and SD (cast: *n* = 5 and titanium: *n* = 10) of the best-fit locations of the Test centroid at 6 components, and their total mean and SD (*n* = 6). As the best-fit matching between Test (cast or titanium frameworks) and its definitive cast was determined with their minimum vertical gap as the parameter, the horizontal distances in *X*, *Y* of the Test are often greater than in *Z*-axis. In total the greatest gap of cast framework was found in *X*-direction (the total mean: 0.0295 mm); however, for the titanium framework the greatest gap was produced in *Y*-direction (the total mean: 0.0041 mm). The total mean vertical gap for cast framework was 0.0134 mm, versus 0.0024 mm for titanium framework.

Table 7 contains the *P* values of unpaired Student's *t*-test on the [Test-Master] centroid location at each component between cast framework and titanium framework. The last column of Table 7 is the unpaired Student's *t*-test in total between cast framework and titanium framework. The statistical significant differences (*P* < 0.05) of the centroid location found with the [Test-Master] difference between the cast framework and the titanium framework are highlighted in bold in Table 7. The centroid location of number 4 component was statistically different in all *X*, *Y*, and *Z* directions between the cast/titanium frameworks. At numbers 2 and 5 components, however, there are no statistical significant differences between the cast/titanium frameworks. The statistical significant differences at numbers 1, 3, and 6 components were found in the *X* and *Z* directions. In total, the statistical difference between the cast framework (0.0134 mm) and the titanium framework (0.0024 mm) was found in the vertical gap (*Z*). 

Table 8 is a comparison of unfixed best-fit matching and the previous method of best-fit matching for the same cast/framework combinations. In the fixed matching coordinate system method, the coordinate origin (0,0, 0) was always located at the number 1 component centroid, the *X*-axis was always through the numbers 1 and 6 (*x*, 0,0) component centroids and the *XY*-plane of the coordinate system was always allied on numbers 1, 6, and 3 (*x*, *y*, 0) component centroids.

The only mean vertical gaps of the [Test-Master] centroid difference generated by the fixed and unfixed best-fit matching coordinate system methods for each specimen are presented in the top portion of Table 8. The second portion of Table 8 contains the mean, SD, and standard error of the centroid [Test-Master] vertical gaps of the cast framework and the titanium framework generated by the fixed and unfixed best-fit matching coordinate system methods. The results demonstrated that the total mean (+SE) vertical gap of the cast framework was 0.021 (+0.004) mm and 0.012 (0.002) mm determined by the fixed and unfixed best-fit matching coordinate system methods, respectively. For the titanium framework they were 0.0037 (+0.0028) mm and 0.0024 (+0.0005) mm, respectively. The paired Student's *t*-test depicted that the differences between the total mean vertical gaps determined by the fixed and unfixed best-fit matching coordinate system methods were statistically significant both for the cast framework (*P* = 0.0321) and the titanium framework (*P* = 0.0256).

## 4. Discussion

Early in the use of endosseous dental implants it was recognized that mechanical overload could have a detrimental effect on the life-time survival of dental implants. It was also recognized that the overload could come from a misfitting prosthesis. It was also acknowledged that strains are transferred to the surrounding bone, when misfitting prostheses are secured [1, 2]. 

However, it has been shown that full-arch cast frameworks do not attain a high level of accuracy, and that clinicians are not capable of detecting the level of misfit during the clinical examination [6].

Since the conventional cast framework distortion occurs during the conventional laboratory fabrication procedures, the elimination of errors caused by expansion of investment and the shrinkage of the alloy should result in a more accurate framework. Other variables that might have affected the fit of a framework include setting expansion of the stone used for the master casts, polymerization shrinkage of the resin framework, and machining accuracy of the components used to fabricate the master cast. A previous study by Jemt demonstrated that a welded one-piece titanium framework has less discrepancy with the fit of the implant frameworks when compared to cast frameworks [11]. What makes the present study different is the fact that the bar is milled from a single block of titanium and not welded smaller components. The mechanism used to digitize the definitive cast and the framework pattern in the present study was completed in the same manner that is done within commercial dental laboratories. Among the reasons that makes this technique successful is the reduction of human and material errors during the fabrication process. 

Previous studies have used a fixed method of assessing vertical gaps between the implant framework and either the abutment or implant replica [11, 15–17]. The fixed method assigns one centroid as the best match and measures the vertical gap at the other centroids. As can be seen in Tables 4 and 5 that method allows for negative readings implying that metal passes through metal. The unfixed method described here uses the centroids to allow the framework to assume the first contact and then measures the vertical gap at the other contact positions (Table 6). This process is used at each centroid to obtain the mean vertical gap of the framework at each mating surface.

Based on the present results, the mean value of the vertical gap between the implant replicas and the milled one-piece titanium implant framework shows a very small number (0.0037 mm or 0.0024 mm by the fixed or unfixed best-fit matching coordinate system methods) relative to the casts frameworks. This value represents the accuracy of fit of these implant frameworks. The present study found that the precision of fit of the milled titanium-fixed complete denture bar was within a range of at least 0.010 mm [10].

**Figure 1:**
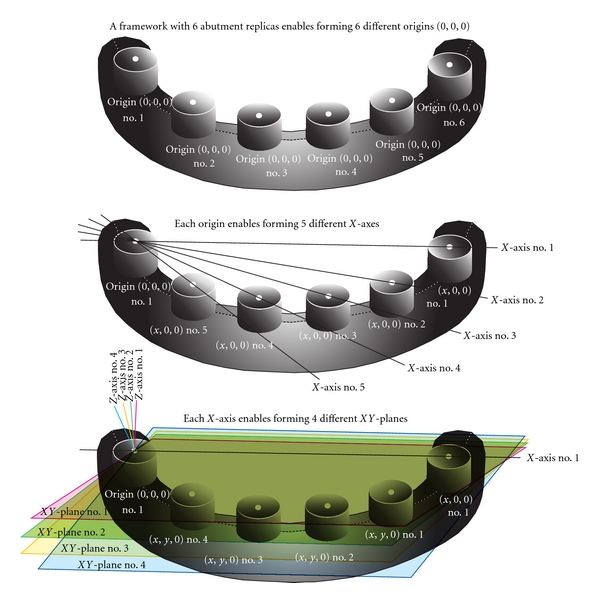
The unfixed matching coordinate system method: 120 possible matching rectangular coordinate systems for a framework containing 6 abutment replicas (possible to form: 6 Origins × 5 *X*-axes × 4 *XY*-planes = 120 coordinate system).

To determine how accurate a fabricated framework is compared with its definitive cast, the 3D differences between two corresponding 3D specific points formed at each paired framework was measured. To represent the 3D specifications of a framework, it is necessary to construct the minimum numbers of three (3) of such specific corresponding points; however, the measurement accuracy for the 3D specification of framework increases with the numbers of each point. In the present study six (6) such specific points were generated from 6 standard abutment replica components obtained from the framework. The vertical best-fit between two 3D entities is achieved when three corresponding specific 3D points on the definitive and its fabricated framework become (0,0, 0), (*x*, 0,0), and (*x*, *y*, 0) and form the identical *x*, *y*-horizontal plane of the matching coordinate system (Figure 1). However, there are total of six such specific points in the present study, and only three of them are needed to form such matching coordinate system. The newly developed mathematical formulae used in the present study allows for matching the definitive/fabricated framework with all 120 possible matching coordinate systems (6 × 5 × 4). The vertical gaps generated by the unfixed best-fit matching coordinate system method were significantly less than the vertical gap generated by the fixed best-fit matching coordinate system method for both cast and titanium frameworks. 

## 5. Conclusion

Computer-aided design/computer-aided machined (CAD/CAM) milled one-piece titanium-fixed complete denture frameworks provided a more accurate precision of fit over that of cast frameworks. Also, the unfixed coordinate system for matching generates better best-fit results compared to match in the fixed coordinate system.

## 6. Clinical Implications

Provided accurate impressions and verified models are used the complications of nonpassive fit of fixed complete denture frameworks can be addressed with the use of a one-piece milled framework.

## Figures and Tables

**Table 1:** The best-fit matching results in *μ*m of the cast frameworks to their respective master casts using the fixed method of alignment. 0,0, 0 represents the matching centroid position, *x*, 0,0 and *x*, *y*, 0 represent the best fit centroids for that position. The final column is the mean vertical gap at that position centroid.

Specimen	Best-fit coordinate system			Cast
	(0,0, 0)	(*x*, 0,0)	(*x*, *y*, 0)	centroid
1	no. 1	no. 2	no. 6	12.6
1	no. 2	no. 6	no. 5	5.8
1	no. 3	no. 2	no. 4	44.4
1	no. 4	no. 2	no. 1	52.7
1	no. 5	no. 2	no. 6	7.6
1	no. 6	no. 2	no. 5	5.8
	Minimum			5.8

2	no. 1	no. 2	no. 6	31.2
2	no. 2	no. 6	no. 5	19.3
2	no. 3	no. 2	no. 5	23.4
2	no. 4	no. 5	no. 3	58.8
2	no. 5	no. 6	no. 2	26.5
2	no. 6	no. 2	no. 3	28.3
	Minimum			19.3

3	no. 1	no. 3	no. 5	15.3
3	no. 2	no. 3	no. 1	223.0
3	no. 3	no. 6	no. 5	18.3
3	no. 4	no. 5	no. 3	49.2
3	no. 5	no. 3	no. 6	16.9
3	no. 6	no. 3	no. 1	12.8
	Minimum			12.8

4	no. 1	no. 2	no. 6	28.2
4	no. 2	no. 6	no. 5	22.7
4	no. 3	no. 2	no. 4	71.0
4	no. 4	no. 2	no. 6	17.2
4	no. 5	no. 6	no. 4	51.9
4	no. 6	no. 2	no. 1	26.3
	Minimum			17.2

5	no. 1	no. 2	no. 6	19.5
5	no. 2	no. 6	no. 3	12.1
5	no. 3	no. 2	no. 6	12.9
5	no. 4	no. 6	no. 3	18.8
5	no. 5	no. 6	no. 3	19.8
5	no. 6	no. 2	no. 3	12.1
	Minimum			12.1

**Table 2:** The best-fit matching results in *μ*m of the number 2 through number 5 cast frameworks to their respective master casts using the fixed method of alignment and the vertical gap at the best positions.

Specimen	Best-fit coordinate system			Cast
	(0,0, 0)	(*x*, 0,0)	(*x*, *y*, 0)	centroid
1	no. 2	no. 6	no. 5	5.8
2	no. 2	no. 6	no. 5	19.3
3	no. 6	no. 3	no. 1	12.8
4	no. 4	no. 2	no. 6	17.2
5	no. 2	no. 6	no. 3	12.1

**Table 3:** The best-fit matching results in *μ*m of the cast frameworks to their respective master casts using the unfixed method of alignment.

Specimen	Best-fit coordinate system			Titan.
	(0,0, 0)	(*x*, 0,0)	(*x*, *y*, 0)	centroid
1	no. 4	no. 6	no. 2	2.0
2	no. 1	no. 4	no. 6	4.4
3	no. 6	no. 3	no. 2	3.8
4	no. 2	no. 5	no. 1	4.8
5	no. 1	no. 3	no. 5	3.0
6	no. 6	no. 2	no. 3	1.5
7	no. 4	no. 1	no. 5	1.2
8	no. 6	no. 3	no. 2	0.9
9	no. 3	no. 1	no. 5	1.0
10	no. 1	no. 2	no. 5	1.9

**Table 4:** The cast [test-master] best-fit differences of each component centroid in *μ*m.

Specimen		Component					
		no. 1	no. 2	no. 3	no. 4	no. 5	no. 6
1	*x *	−21.6	−44.6	107.2	−53.0	81.0	0.0
1	*y *	118.0	11.8	−10.6	−28.2	−43.4	0.0
1	*z*	23.4	0.6	7.1	0.0	2.8	0.9

2	*x *	98.5	0.0	52.9	−80.7	−42.4	89.2
2	*y *	−87.9	0.0	28.4	18.1	19.6	−23.7
2	*z*	44.1	0.8	14.3	55.2	0.0	1.2

3	*x *	23.9	98.7	−36.0	−17.9	−53.0	0.0
3	*y *	148.1	48.6	18.0	−34.4	63.8	0.0
3	*z*	0.0	30.2	0.0	42.4	4.1	0.0

4	*x *	140.8	31.5	79.6	0.0	18.4	69.0
4	*y *	8.6	9.7	−89.8	0.0	8.5	107.4
4	*z*	54.0	4.6	23.4	5.2	15.9	0.0

5	*x *	63.4	0.0	126.8	−9.4	98.3	65.9
5	*y *	147.9	0.0	89.4	−43.4	−71.2	−17.4
5	*z*	21.7	0.0	3.0	23.9	23.4	0.3

Mean	*x *	61.0	17.1	66.1	−32.2	20.5	44.8
Mean	*y *	66.9	14.0	7.1	−17.6	−4.6	13.3
Mean	*z*	28.6	7.2	9.6	25.4	9.2	0.5

SD	*x *	63.2	53.1	63.5	33.7	69.1	41.9
SD	*y *	103.8	20.1	65.3	25.7	53.3	53.7
SD	*z*	21.1	13.0	9.4	23.6	10.0	0.5

**Table 5:** The titanium [Test-Master] best-fit differences of each component centroid in *μ*m.

Specimen		Component					
		no. 1	no. 2	no. 3	no. 4	no. 5	no. 6
1	*x *	−1.9	−3.2	14.3	0.0	−5.5	−0.9
1	*y *	20.1	11.9	14.9	0.0	−0.5	1.1
1	*z*	4.5	0.0	1.4	0.5	5.2	0.4

2	*x *	0.0	19.3	−6.7	22.1	−2.0	18.2
2	*y *	0.0	7.8	2.6	12.7	1.5	−29.8
2	*z*	0.5	4.8	8.5	0.0	10.8	1.6

3	*x *	4.2	1.9	−26.3	9.6	5.6	0.0
3	*y *	11.6	3.5	13.1	8.3	6.9	0.0
3	*z*	0.6	0.1	0.0	16.4	6.0	0.0

4	*x *	17.1	0.0	24.6	16.1	−2.1	−28.4
4	*y *	−16.2	0.0	−11.4	−15.2	0.0	26.9
4	*z*	0.0	0.0	7.9	11.0	0.0	9.6

5	*x *	0.0	9.8	−3.8	−3.7	21.2	−7.3
5	*y *	0.0	20.2	−4.5	−3.9	3.2	31.9
5	*z*	0.3	3.6	0.3	9.4	0.0	4.3

6	*x *	−18.0	−21.1	−16.6	−12.5	−8.5	0.0
6	*y *	14.6	5.7	17.9	18.3	10.2	0.0
6	*z*	1.4	0.0	0.4	6.5	0.7	0.1

7	*x *	5.0	8.4	8.8	0.0	5.8	12.4
7	*y *	2.9	4.9	3.2	0.0	−0.1	−3.7
7	*z*	0.0	4.0	2.0	0.1	0.0	1.0

8	*x *	−23.5	−19.4	−10.1	0.7	−4.8	0.0
8	*y *	17.9	4.1	5.1	1.6	1.9	0.0
8	*z*	0.0	0.0	0.2	4.0	1.2	0.2

9	*x *	0.2	0.1	0.0	−7.1	2.2	4.1
9	*y *	0.2	1.4	0.0	3.1	3.3	0.8
9	*z*	0.0	2.1	0.0	2.4	0.0	1.1

10	*x *	0.0	−2.4	−2.1	−19.5	−8.7	−0.1
10	*y *	0.0	−3.5	−3.8	1.8	10.1	13.8
10	*z*	0.0	0.1	1.8	8.4	0.0	0.9

Mean	*x *	−1.7	−0.7	−1.8	0.6	0.3	−0.2
Mean	*y *	5.1	5.6	3.7	2.7	3.6	4.1
Mean	*z*	0.7	1.5	2.2	5.9	2.4	1.9

SD	*x *	11.5	12.4	14.9	12.6	9.0	12.3
SD	*y *	11.0	6.6	9.3	9.2	4.0	17.2
SD	*z*	1.4	2.0	3.2	5.5	3.7	3.0

**Table:** (a)

Cast (*n* = 5) Component		Test-ref no. 1	Test-ref no. 2	Test-ref no. 3	Test-ref no. 4	Test-ref no. 5	Test-ref no. 6	Test-ref Total (*n* = 6)
*X *	Mean	61.0	17.1	66.1	−32.2	20.5	44.8	29.5
*Y *	Mean	66.9	14.0	7.1	−17.6	−4.6	13.3	13.2
*Z*	Mean	28.6	7.2	9.6	25.4	9.2	0.5	13.4

*X *	SD	63.2	53.1	63.5	33.7	69.1	41.9	36.4
*Y *	SD	103.8	20.1	65.3	25.7	53.3	53.7	28.9
*Z*	SD	21.1	13.0	9.4	23.6	10.0	0.5	11.1

**Table:** (b)

Cast (*n* = 10) Component		Test-ref no. 1	Test-ref no. 2	Test-ref no. 3	Test-ref no. 4	Test-ref no. 5	Test-ref no. 6	Test-ref Total (*n* = 6)
*X *	Mean	−1.7	−0.7	−1.8	0.6	0.3	−0.2	−0.6
*Y *	Mean	5.1	5.6	3.7	2.7	3.6	4.1	4.1
*Z*	Mean	0.7	1.5	2.2	5.9	2.4	1.9	2.4

*X *	SD	11.5	12.4	14.9	12.6	9.0	12.3	1.0
*Y *	SD	11.0	6.6	9.3	9.2	4.0	17.2	1.1
*Z*	SD	1.4	2.0	3.2	5.5	3.7	3.0	1.8

**Table 7:** *P* values of unpaired Student's *t*-test on the [Test-ref] centroid location differences in *μ*m at each component of Cast framework and titanium framework. The rightest column presents the unpaired Student's test of in total between cast/titanium frameworks.

	no. 1	no. 2	no. 3	no. 4	no. 5	no. 6	Total
*X *	**0.0077 **	0.3167	**0.0056 **	**0.0154 **	0.3633	**0.0065 **	0.0698
*Y *	0.0749	0.2381	0.8706	**0.0397 **	0.6239	0.6212	0.4616
*Z*	**0.0008**	0.1767	**0.0406**	**0.0236**	0.702	0.3107	**0.0373**

**Table:** (a)

Centroid *Z* gap data base	Fixed cast	Unfixed cast	Fixed titan.	Unfixed titan.
1	12.1	5.8	3.2	2.0
2	31.7	19.3	8.7	4.4
3	12.8	12.8	3.9	3.8
4	29.9	17.2	8.6	4.8
5	18.5	12.1	4.4	3.0
6			1.9	1.5
7			2.1	1.2
8			0.9	0.9
9			1.3	0.9
10			2.3	1.9

**Table:** (b) Descriptive statistics

	Count	Mean	Std. dev.	Std. error
Fixed cast	5	21.00	9.306	4.162
Unfixed cast	5	13.44	5.221	2.335

Fixed titan.	10	3.73	2.812	0.889
Unfixed titan.	10	2.44	1.462	0.462

**Table:** (c) Paired *t*-test: hypothesized difference = 0

	Mean diff.	DF	*t* value	*P* value
Fixed cast; unfixed cast	7.56	4	3.225	0.0321
Fixed titan; unfixed titan.	1.29	9	2.671	0.0256
